# Decision support system through automatic algorithms and electronic request in diagnosis of anaemia for primary care patients

**DOI:** 10.11613/BM.2021.020702

**Published:** 2021-04-15

**Authors:** Enrique Rodriguez-Borja, Adela Pozo-Giraldez, Macarena Díaz-Gimenez, Ausias Hervas-Romero, Africa Corchon-Peyrallo, Inmaculada Vinyals-Bellido, Arturo Carratala Calvo

**Affiliations:** Laboratory of Biochemistry, Valencia University Clinic Hospital, Valencia, Spain

**Keywords:** algorithms, anaemia, diagnosis, clinical laboratory information systems, cost – effectiveness, referral and consultation

## Abstract

**Introduction:**

An appropriate management of anaemia laboratory tests is crucial for a correct diagnosis and treatment. A non-sequential “shotgun” approach (where every anaemia related test is ordered) causes workload and cost increases and could be potentially harmful. We have implemented a Decision Support System through our laboratory information system (LIMS) based on reflexive algorithms and automatic generation of interpretative reports specifically in diagnosis of anaemia for primary care patients.

**Materials and methods:**

When a request contained an “Anaemia Suspicion Study” profile, more than twenty automatic reflexive rules were activated in our LIMS based upon laboratory results. These rules normally involved the addition of reflexive tests. A final report was automatically generated for each interpretation which was always reviewed for their validity by two staff pathologists. We measured the impact of this system in the ordering of most common anaemia related tests and if a proper treatment was established based on the interpretive report.

**Results:**

From all the studies performed, only 12% were positive being “iron deficiency” and “anaemia of chronic disease” the most frequent causes, 62% and 17%, respectively. Proper treatment was established in 88% of these anaemic patients. Total iron, transferrin, ferritin, folate and vitamin B12 demand decreased substantially after implementation representing a cost reduction of 40% only for these five tests.

**Conclusions:**

Our system has easily improved patient outcomes, advising on individual clinical cases. We have also noticeably reduced the number of over-requested tests and laboratory costs.

## Introduction

Anaemia is the most common haematological disorder that affects the population in both developing and developed countries. Anaemia adversely affects patient´s quality of life and is a condition associated with higher morbidity and mortality (*1*). Furthermore, it is a common problem in primary care and generally constitutes an important challenge to health-care systems in terms of diagnosis and management (*2*).

The proper diagnosis of anaemia is strongly based on laboratory results. In fact, it is considered to be present if the haemoglobin (Hb) concentration is below the lower limit of the 95% reference interval for the individual´s age and sex (*3*). Several algorithms on the Hb result have been proposed as a guide to better characterize the disorder. In most of them, red cell volume (RCV or MCV) has been traditionally used to classify and define the type of anaemia (microcytic, normocytic and macrocytic). Although other discriminant algorithms have been recently proposed including new red blood cells (RBC) parameters, the anaemia classification system based on MCV proposed almost ninety years ago by Maxwell Wintrobe, remains a useful tool for physicians (*4*-*6*).

On balance, an appropriate management of anaemia laboratory tests is crucial for a correct diagnosis and treatment. The cost of these tests may be small in comparison to the costs resulting from delayed diagnosis (additional visit, referrals, *etc.*) and that might be the main reason why General Practitioners (GPs) often elect to order every possible anaemia related test during the initial visit in primary care environments. Far from being efficient, this non-sequential “shotgun” approach causes workload and cost increases and could be potentially harmful due to diagnostic confusion or forgotten tests (*7*).

In fact, Salinas *et al.* recently showed that the demand for and expenditure on anaemia related tests in primary care in Spain is markedly elevated with significant regional differences compared to previous studies performed a couple of years earlier (*8*). This robust study was conducted analysing data coming from a total of 110 laboratories all over the country, corresponding to a catchment area of nearly 28 million inhabitants (60% of the Spanish population). Their conclusion emphasized the necessity of further studies focused on how laboratories could help GPs in anaemia test management, through computer-aided algorithms (*9*).

Clinical laboratory consultation has been described as one of the most promising solutions to inappropriate utilization of laboratory services (*10*). Nowadays, clinical laboratory consultants could be able to fill the gap between raw laboratory data and information easily understandable to the clinicians, adopting a more active role.

In 1999, Smith *et al.* reported the use of a ‘‘Decision Support System’’ which interacted with providers to assist them in laboratory test selection and result interpretation. Despite the good results in terms of fewer tests being ordered during a diagnostic evaluation, fewer blood samples being collected from the patient and a shortening in the time to diagnosis, there was no data on whether the information reported by this automatic system was correct or not (*11*). It has been estimated that automatic algorithms for diagnosis or treatment are not 100% effective in terms of test selection and interpretation, so the presence of an expert consultant during this process would be highly recommended.

In 2000, Laposata and his associates, who are considered the pioneers of laboratory consultation services, instituted formal sign out rounds with the combination of reflexive test algorithms and the generation of interpretative reports in assorted areas like coagulation, haemoglobin/anaemia and autoimmune diseases. They advocated for a substantial improvement in the model of laboratory test ordering and interpretation. In this model, laboratory experts regularly supply valuable information regarding test selection and result interpretation to the ordering clinicians. By means of this arrangement, they showed an improvement in the quality of care, a reduction in the cost per case by decreasing the time to diagnosis, the number of tests ordered and the number of patient visit (*12*).

Two years later, these same authors expressed their concerns regarding computerized “intelligent” laboratory information systems which could guide ordering physicians in terms of requesting advice on the selection and interpretation on laboratory tests. They stated that given that the appropriateness of a particular test could vary tremendously because of the different clinical situations in which this parameter might be ordered, the advice of an “intelligent” software could be extremely difficult to implement (*13*).

The main aim of our study was to combine both perspectives, implementing a computerized “Decision Support System” through our computer physician order entry (CPOE) and laboratory information system (LIMS), that not only could identify the clinical situation of the patient but also involve an expert laboratory professional in each clinical case for the final review and verification. This system would be based on reflexive algorithms and automatic generation of interpretative reports and would be applied specifically in diagnosis of anaemia for primary care patients.

Additionally, we wanted to measure the impact of this system in the ordering of most common anaemia related tests such as ferritin, transferrin, total iron, folate and vitamin B12 in order to know if it is also a good strategy for test management and cost savings.

And finally, we wanted to determine if the initiative was clinically useful for our GPs in terms of establishing proper treatments in these patients based on the interpretive reports given by the laboratory.

## Materials and methods

### Subjects

This prospective cohort study for primary care patients was conducted from January 2018 to December 2019 in the Laboratory of Biochemistry and Molecular Pathology of University Clinic Hospital in Valencia (Spain). This hospital is a tertiary referral center that serves around 360,000 people in Valencia´s metropolitan area, the third biggest city in the country by population. All the laboratory requests are ordered via a CPOE system directly linked to our LIMS, Gestlab (Cointec Ingenieros, Inc., Alicante, Spain) since 2011.

Our “Primary Care Anaemia Algorithm Strategy” (Supplementary data) was implemented in July 1st 2018. We collected data over a total 2-year period (January 2018 to December 2019) and divided into two phases for comparison purposes:

Phase PRE: 6-month period before algorithm implementation (from January 2018 to June 2018).

Phase POST: 6-month period after one year of the intervention (from July 2019 to December 2019).

All the data were retrieved from our LIMS and our electronic medical records. Our study was approved by Valencia´s Clinic Hospital Ethics Committee.

### Methods

We redesigned our electronic panel formulary for primary care introducing three new clinical profiles or “workups” available for all our GPs that could be selected through a tick-box system. The first profile was called “Anaemia Suspicion Study” and included a complete blood count (CBC) and an order for an extra serum tube venipuncture (in case any serum test was ordered in the request). When this profile was ordered, providers were always asked to answer two simple free-text compulsory questions regarding suspected diagnosis and the patient’s main symptomatology. The second profile was called “Microcytic Anaemia Follow Up” and included a CBC and ferritin test. Finally, the third profile was called “Normo/Macrocytic Anaemia Follow Up” and just included a CBC. A restriction rule through our CPOE made it impossible to order more than one of these profiles in the same request.

Anaemia related tests such as ferritin, transferrin, total iron, folate and vitamin B12 were not included directly in the panel as tick-boxes, but they could be added to the request without any limitation through a search engine function in our CPOE system.

Our “Primary Care Anaemia Algorithm Strategy” was fully agreed between Biochemistry and Haematology Laboratories before its implementation. When a request contained an “Anaemia Suspicion Study” profile, more than twenty automatic reflexive rules were activated in our LIMS based upon laboratory results and reference values. These rules normally involved the addition of reflexive tests. A final report was automatically generated for each interpretation. These reports were always reviewed for their validity by two staff pathologists according to the provided results and the patient’s medical records. These consultors were free to add extra tests and/or introduce specific comments to the automatic report in light of the information obtained and they didn´t represent any additional cost in terms of time and/or input into the process. If anaemia was not detected in the patient (normal haemoglobin levels for age and sex) an automatic result was reported and no more tests added (Report 0): *“The patient is not anaemic. Further studies are not necessary”*. Previously to its implementation, the Primary Care Anaemia Algorithm Strategy was fully explained to each and every GP in our department via e-mail and face-to-face meetings with the different primary care centers. Several suggestions coming from our GPs were finally adopted (*e.g.* serum transferrin receptor test, referral to haematologist).

Time of samples collection was always between 8:00–9:30 AM for all the requests of the study. So, thresholds used for total serum iron were those regarding that specific time of collection.

We collected all the number of tests performed for total iron, transferrin, ferritin, folate and vitamin B12, as well as the three profiles mentioned above, for the timeframe of the study. The number of tests that were performed and their change in demand was studied as a ratio of total tests per total primary care requests (in percentage %).

Furthermore, the laboratory cost in euros (€) was calculated according to Valencia´s Autonomic Health Tax Law for each test for the period before pre-intervention (Phase PRE) *vs* the 6-month period after one year of the intervention (Phase POST). Cost reductions as percentage were calculated comparing both periods of time.

For each “Anaemia Suspicion Study” ordered, the data from the final report from the laboratory were collected. Additionally, if the result was consulted by the GPs in a week (through our results webpage) and if a proper treatment was established according to Laboratory recommendations and Department guidelines, the data were also retrieved.

### Statistical analysis

The Kolmogorov-Smirnov test was used to assess the normality of distribution of investigated parameters. All parameters in our study were distributed normally. Data were expressed as mean ± standard deviation. Differences were tested by paired two-tailed t-test. The values P < 0.05 were considered statistically significant. Statistical analysis was done using IBM SPSS Statistics for Windows, Version 26.0. (IBM, Armonk, NY, USA).

## Results

We performed 1994 “Anaemia Suspicion” studies, 10,026 “Microcytic Anaemia Follow Up” studies and 745 “Normo/Macrocytic Anaemia Follow Up” studies. From all the “Anaemia Suspicion” studies, 88% were negative and patients were not anaemic (Table 1). The most frequent causes of anaemia were “iron deficiency” and “anaemia of chronic disease”. Altogether 98% of the reports for a confirmed anaemia were consulted by GPs within a week of receiving the result. Proper treatment was established in 88% of these patients.

**Table 1:** Positive “Anaemia Suspicion Study” results for our Primary Care Algorithm Strategy from July 2018 to December 2019

**Results**	Automatic report on LIMS*	**Number of cases**	**Anaemia cases consulted**	**Anaemia cases with proper treatment**
Iron deficiency	1.1, 1.2	149	147	131
CDA: Renal insufficiency	4.1	22	22	19
CDA: Liver disease	4.2	4	3	3
CDA: Thyroid disease	4.3	4	4	4
CDA: Inflammatory	4.4	10	8	8
Possible posthaemorrhagic anaemia	2.1, 2.2, 3.1 & 3.2	17	17	13
Refer to haematologist	1.4, 2.4 & 3.4	14	14	14
Megaloblastic anaemia	3.5	8	8	7
Haemoglobinopathies	1.3	7	6	6
Haemolytic anaemia	2.3 & 3.3	3	3	3
Anaemia of chronic disease with iron deficiency	1.5	2	2	2
Total anaemia cases determined	NA	240	234	210
*Automatic Report on LIMS refers to the comments from algorithm in Supplementary data. CDA - chronic disease anaemia. NA - non applicable. LIMS - laboratory information system.				

We found a significantly lower level of test demand in post intervention phase as compared to pre intervention phase for all the tests studied (except the implemented clinical profiles) (Table 2).

**Table 2:** Tests ratios per total primary care requests in percentage (%) in Phase PRE – intervention (from January 2018 to June 2018) and Phase POST – intervention (from July 2019 to December 2019)

**Test**	**Phase PRE (%) (mean ± SD)**	**Phase POST (%) (mean ± SD)**	**P**
Total iron	26.7 ± 0.7	12.2 ± 0.6	< 0.001
Transferrin	9.0 ± 0.1	5.1 ± 0.4	< 0.001
Ferritin	26.0 ± 0.3	19.2 ± 0.8	< 0.001
Folate	11.3 ± 0.6	6.6 ± 0.3	< 0.001
Vitamin B12	13.7 ± 0.7	8.6 ± 0.5	< 0.001
“Anaemia Suspicion Study”	NA	0.9 ± 0.2	NA
“Microcytic Anaemia Follow Up”	NA	6.5 ± 0.3	NA
“Normo/Macrocytic Anaemia Follow Up”	NA	0.5 ± 0.1	NA
Tests ratios are expressed as mean ± standard deviation (SD). Differences were tested by paired two-tailed t-test. NA - not applicable.			

Regarding cost reductions after implementing the algorithm, and taking into account all these results, a total reduction of 40% was estimated between pre and post intervention phases. Total iron was the test with the higher cost reduction in percentage while ferritin had the lower cost reduction. This represents a monthly reduction of 33,454 € for these five tests (Table 3).

**Table 3:** Total test cost in Euros (€) in Phase PRE – intervention (from January 2018 to June 2018) and Phase POST – intervention (from July 2019 to December 2019) and cost reduction in percentage (%)

**Test**	**Phase PRE (€)**	**Phase POST (€)**	**Cost reduction (%)**
Total iron	18,169	7921	57
Transferrin	66,987	35,057	48
Ferritin	165,016	113,711	31
Folate	113,718	62,686	45
Vitamin B12	137,147	81,390	41
Total	501,487	300,765	40

## Discussion

We have successfully implemented a complete “Decision Support System” that is able to identify the clinical situation of the patient through our CPOE and LIMS for our primary care providers. For all the “Anaemia Suspicion” studies ordered, our system automatically generates a final report based on the results obtained.

Most of the “Anaemia Suspicion Studies” ordered came back negative and patients were not anaemic despite the initial suspicion of our GPs. As expected from primary care, the most frequent causes of anaemia were “iron deficiency”, “anaemia of chronic disease” and “possible posthaemorrhagic anaemia”. Despite the very low rates of “Anaemia Suspicion Study”, “Microcytic Anaemia Follow Up” and “Normo/Macrocytic Anaemia Follow Up” profiles in our daily primary care requests our results have shown a substantial rate of decrease measured for anaemia related tests (total iron, transferrin, ferritin, folate and vitamin B12).

In particular, the noteworthy decrease and current evidence in the appropriate utilization of total iron and transferrin, show that these tests were over requested in our department. In fact, total iron and transferrin have been considered both redundant in iron deficiency anaemia and their values may overlap between healthy and iron deficient population. Some protocols include adding these tests only if serum ferritin is not diagnostically relevant (when its values are normal or increased) in patients with concomitant chronic inflammation (*14*). Although we have also observed a decline in its demand, it has not been as significant as total iron or transferrin given that ferritin was also included in the “Microcytic Anaemia Follow Up” clinical profile. This specific profile is finally ordered around six times more than “Anaemia Suspicion Study” profile since there are much more patients with a fully diagnosed microcytic anaemia being followed up with than patients with suspected anaemia.

Regarding folate and vitamin B12, our results suggest they were also inappropriately over requested. Rates of decrease have been higher for folate rather than vitamin B12. This could be explained by the fact that neuropsychiatric symptoms in elderly people is one of the additional indications for vitamin B12 testing in primary care settings prior to neurologist referral (*15*). Furthermore, folate determination in primary care has been considered of little clinical significance in those patients without known risk factors for folate deficiency (*16*).

Our “Primary Care Anaemia Algorithm Strategy” has been highly efficient in terms of clinical management of suspected anaemia and it has been clinically useful since a great majority of the reports were consulted within a week of receiving the results enabling a proper treatment establishment. One of the major strengths of our study is that we have been able to improve patient care through our interpretations; to easily influence patient outcome and to notably reduce costs using an “algorithmic – sequential” approach for diagnosing anaemia.

We have noticed that sometimes our algorithm could not offer a direct explanation for some difficult cases, mostly related with anaemia of chronic disease in pluripathological patients. Though few in number, in these cases, the presence of an expert consultant has been really useful to overcome the limitations of the computerized rules (*e.g.* adding new tests, introducing additional comments). In fact, it is obvious to us that just relying on automatic algorithms could be insufficient and unsatisfactory, and we strongly recommend the participation of a professional in order to ensure the proper functioning of the process.

The interpretation of abnormal test results personally by the laboratory specialist based on the clinical judgment has been recognized as a useful tool to improve the process of patient’s diagnosis and treatment (*17*). Several studies performed in Netherlands have showed that reflective testing, as a form of consultation, adds value in the service of clinical laboratory to primary health care providers and is generally welcomed by the doctors (*18*, *19*). The automated generation of interpretative comments based on results normally implies the application of expert systems. Several approaches have been suggested: from basic algorithms that are sequentially executed (like our study), through Bayesian reasoning (where probability of a diagnosis is calculated given the patient variables) to more complex neural networks (where the larger the amount of data available, the more efficient the outcomes are) (*20*).

Gunčar *et al*. recently built two models to predict a haematological disease using machine learning algorithms based on laboratory results (*21*). Both predictive models produced good results in terms of accuracy being on par with that of haematology specialists, but they were unable to make a complete differential diagnosis of anaemia cases and more importantly they required a full data matrix of test results for each patient in order to make an accurate prediction. As far as we know, these models only work backwards (they don’t perform tests sequentially based on previous results), so from a laboratory point of view, the potential cost savings related to test management remain unclear. Although a promising medical tool, the advice of “intelligent” software in this field is far from being fully developed and the involvement of a laboratory consultant is still necessary to successfully integrate test results and available clinical information.

Our “algorithmic – sequential” approach could be easily adapted with little effort by any clinical laboratory using a LIMS that allows normal reflexive testing. Moving from classical “organ-based” profiles toward “disease-”, “symptom-”, or “question-specific” profiles could be a promising opportunity for laboratories to offer a default test selection for a specific clinical situation that can be supplemented, if necessary, through algorithms (*22*). Additionally, our expert application fulfils the gap of adding interpretative customized comments and appropriate tests while dealing with the increasing number and complexity of daily requests.

By means of this new procedure we have generally improved patient outcomes, advising on individual clinical cases and potential treatments. We have also noticeably reduced laboratory costs, decreasing the number of over-requested tests and indirectly we have strengthened the position of laboratory professionals through automatic consultation.
